# Prevalence of psychomorbidity among patients with chronic cough

**DOI:** 10.1186/1745-9974-2-4

**Published:** 2006-06-16

**Authors:** Lorcan PA McGarvey, Carol Carton, Lucy A Gamble, Liam G Heaney, Richard Shepherd, Madeline Ennis, Joseph MacMahon

**Affiliations:** 1 Department of Respiratory Medicine, Belfast City Hospital, N. Ireland, UK; 2 Department of Clinical Psychology, Belfast City Hospital, N. Ireland, UK; 3 Department of Clinical Biochemistry, The Queen's University of Belfast, N. Ireland, UK

## Abstract

**Background:**

Chronic cough may cause significant emotional distress and although patients are not routinely assessed for co-existent psychomorbidity, a cough that is refractory to any treatment is sometimes suspected to be functional in origin. It is not known if patients with chronic cough referred for specialist evaluation have emotional impairment but failure to recognise this may influence treatment outcomes. In this cross-sectional study, levels of psychomorbidity were measured in patients referred to a specialist cough clinic.

**Methods:**

Fifty-seven patients (40 female), mean age 47.5 (14.3) years referred for specialist evaluation of chronic cough (mean cough duration 69.2 (78.5) months) completed the Hospital Anxiety and Depression (HAD) scale, State Trait Anxiety Inventory (STAI) and the Crown Crisp Experiential Index (CCEI) at initial clinic presentation.

Subjects then underwent a comprehensive diagnostic evaluation, after which they were classified as either treated cough (TC) or idiopathic cough (IC). Questionnaire scores were compared between TC (n = 42) and IC (n = 15).

**Results:**

Using the HAD scale, 33% of all cough patients were identified as anxious, while 16% experienced depression. The STAI scores suggested moderate or high trait anxiety in 48% of all coughers. Trait anxiety was significantly higher among TC (p < 0.001) and IC patients (p = 0.004) compared to a healthy adult population. On the CCEI, mean scores on the phobic anxiety, somatisation, depression, and obsession subscales were significantly higher among all cough patients than the published mean scores for healthy controls. Only state anxiety was significantly higher in IC patients compared with TC patients (p < 0.05).

**Conclusion:**

Patients with chronic cough appear to have increased levels of emotional upset although psychological questionnaires do not readily distinguish between idiopathic coughers and those successfully treated.

## Background

Chronic cough is a common and disruptive symptom, which impacts adversely on a patient's quality of life [1]. Individuals with a persistent cough frequently report exhaustion, sleep deprivation and social withdrawal and it is reasonable to expect an increased level of emotional distress in this patient group. However, patients evaluated for chronic cough are not routinely assessed for concurrent psychomorbidity. A few studies have suggested a relationship between cough and emotional distress. In a community-based study, Ludviksdottir *et al *reported that persistent coughing was significantly associated with anxiety, although study participants were not representative of those typically referred for evaluation of chronic cough [2]. Hutchings and co-workers reported that individuals with obsessive traits were unable to voluntarily suppress experimentally induced cough [3]. Recently, a high prevalence of depressive symptoms in patients with chronic cough has been reported [4]

Although management strategies for chronic cough are often successful [5], in some circumstances, coughing may persist in the absence of an identifiable cause and despite extended trials of empirical therapy [6]. Such individuals have been classified as having an idiopathic cough (IC). Although sometimes suspected of having a functional disorder, it is not known if idiopathic coughers have a different range and severity of psychological distress compared to those with treatable cough.

Therefore, the aims of this study were to

1. determine the levels and range of psychomorbidity in patients referred to a specialist cough clinic

2. determine whether differences in psychomorbidity exist between patients subsequently diagnosed as idiopathic coughers and those in whom a cause for cough is identified and successfully treated.

## Methods

### Subjects

Patients with non-productive cough persisting for more than eight weeks as their sole respiratory symptom were recruited from the cough clinic at Belfast City Hospital. All patients had been physician referred, aged between 18 and 80 years, were lifetime non-smokers, and had a normal chest radiograph and spirometry. Patients with a previous history of chest disease, any systemic disease, an upper respiratory tract infection (URTI) within the preceding 8 weeks or those taking angiotensin converting enzyme inhibitors (ACE-Is) were excluded. No patient had a history of previous psychiatric disease. The Research Ethics Committee of the Queen's University of Belfast approved the study and written informed consent was obtained from all subjects.

### Psychological measurements

Each patient was asked to complete the following three questionnaires at the first outpatient visit;

Hospital Anxiety and Depression (HAD) scale [7],

State Trait Anxiety Inventory (STAI) [8]

Crown Crisp Experiential Index (CCEI) [9].

These three validated questionnaires were chosen because they were short, self report assessment instruments, and each had published healthy and patient control scores for comparison. Further information regarding each questionnaire is detailed below;

The HAD scale is a well validated 14 item questionnaire giving a rating for a person on anxiety and depression subscales which score from 0 – 21. A score of 8 – 10 is borderline and 11 or greater indicates probable disorder.

The STAI measures the underlying tendency to anxiety in the individual (trait) and how anxious they are at that present moment (state). State anxiety is believed to reflect a transitory emotional state that is characterised by subjective, consciously perceived feelings of tension and apprehension. State anxiety may fluctuate over time and can vary in intensity. In contrast, trait anxiety refers to the general tendency of the individual to respond with anxiety to perceived threats in the environment. Norms have been established and published for a population of healthy adults and for general medical and surgical patients with and without psychiatric disorders [8]. Low, moderate and high anxiety categories for scores on the STAI questionnaire have been established by Auerbach and were used for comparison in this study [10].

The CCEI is a standardised self rating inventory which scores on each of six scales, measuring free floating anxiety, phobic anxiety, obsessionality, somatic anxiety, depression and hysteria. It is designed to obtain a quick approximation to the diagnostic information that would be gained from a formal psychiatric interview. CCEI scores for healthy controls and a group of psychiatric outpatients are available [9]. Participants were also asked to record their cough symptom severity using a visual analogue scale (VAS).

### Diagnostic evaluation

All patients underwent evaluation for cough based on a comprehensive diagnostic protocol, the details of which have been published elsewhere [6]. In brief, after history and physical examination, chest radiograph and spirometry were arranged in all patients. Where indicated, 24 hour oesophageal pH monitoring and/or bronchoprovocation challenge testing were requested. Suspected asthmatic cough or gastro-oesophageal reflux associated cough was treated according to our established management protocol. Patients with normal spirometry and no evidence of bronchial hypereactivity received two weeks of oral prednisolone to exclude a steroid responsive cough. Patients with persisting upper airway symptoms despite intensive nasal therapy underwent formal ear, nose and throat (ENT) assessment and/or CT scan of sinuses. Diagnoses were considered on the basis of a consistent history and/or investigation but were only accepted as contributing to cough when the patient reported satisfactory improvement or complete resolution after a period of diagnosis – specific therapy. A satisfactory improvement was recorded when the patient reported that the cough had subsided to the extent that it was no longer troublesome.

### Data analysis

Descriptive statistics for the standardised measures of the psychoneurotic symptoms were used. Values are given as mean (standard deviation) unless otherwise stated. The range is given where appropriate. As the questionnaire scores for the cough patients were normally distributed, comparisons between treated cough and idiopathic patients were made using unpaired t-Tests. Differences between means of published healthy control population and cough patients were calculated using independent sample t tests. A Pearson correlation coefficient matrix was constructed for assessment of both internal consistency and inter-correlation for the scales. A p value of < 0.05 was considered statistically significant.

## Results

Fifty-seven unselected patients (40 female) were recruited and completed the questionnaires. The mean age was 47.5 (14.3) years and patients had been coughing for 69.2 (78.5) months. The range of cough duration was from 2 months to 240 months. Seventeen (29.8%) patients volunteered that stressful situations precipitated their cough. Two distinct groups were identified using the diagnostic protocol, one where a cause for cough was identified and successfully treated (TC) (n = 42 patients) and the other, idiopathic (IC) (n = 15 patients). Both groups were matched for cough severity on VAS assessment. The causes of cough identified were as follows; cough variant asthma (CVA), n = 15, postnasal drip syndrome (PNDS), n = 10, gastro-oesophageal reflux disease (GORD), n = 11, and dual aetiologies, n = 6.

### HAD scale

The means and standard deviations for the HAD are displayed in table 1. There are no normal population values for the HAD scale, but there are widely accepted cut off values which have been validated in several studies [11]. With these cut off values, 21% of cough patients scored as borderline anxiety cases (score >8 and < 11) and 12.3% experienced clinically important symptoms (score ≥ 11). On the HAD depression subscale, 10.5% were classified as having borderline depression and 5.3% with clinically important symptoms (scores ≥ 11).

**Table 1:** Mean (SD) psychological questionnaire scores for cough patients and published controls [8]

***Psychological measure***	***All cough (n = 57)***	***Idiopathic cough (n = 15)***	Treated cough ***(n = 42)***	***Normal adult population***^8 ^***(n = 694)***	***Medical/surgical patients without psychiatric disorder***^8 ^***(n = 110)***	***Medical/surgical patients with psychiatric disorder***^8 ^***(n = 34)***
***HAD scale***						
*Anxiety*	*6.4 (4.4)*	*5.23 (3.6)*	*6.7 (4.7)*	-	-	-
*Depression*	*3.8 (3.8)*	*3.9 (3.9)*	*3.8 (3.7)*	-	-	-
						
***STAI***						
*State*	*32.3 (8.8)*	*36.5 (9.5)*	*30.9(8.2)*	*33.40 (9.50)*	*42.7 (13.8)*	*42.4 (15.7)*
*Trait*	38.9 (11.3)*	39.15 (8.8)**	38.9 (12.2)***	*32.8 (8.3)*	*41.3 (12.5)*	*44.6 (14.1)*

Values given as mean (SD)

* p < 0.001 All cough versus normal adult population [8]

** p = 0.004 Idiopathic cough versus normal adult population [8]

*** p < 0.001 Treated cough versus normal adult population [8]

**HAD **– Hospital Anxiety and Depression scale, **STAI **– State-Trait Anxiety Inventory

### STAI

Using the categories established by Auerbach [10], for trait anxiety, moderate and high levels of anxiety were identified in 44.2% and 3.8% of subjects respectively. On the state anxiety scale, no patient achieved a high anxiety score, although moderate anxiety was identified in 28% of patients. The remaining patients (72%) could be classified as low state anxiety.

The means and standard deviations for scores on the STAI for the study population compared with norms established by Spielberger [8] are displayed in table 1. Trait anxiety was significantly higher among all coughers compared to the healthy adult population (p < 0.001). This was the case for both idiopathic (p = 0.004) and successfully treated coughers (p < 0.001). However, there was no significant difference in trait anxiety scores between all coughers and the published medical and surgical reference population (p = 0.23)[8]. There was no significant difference between state anxiety scores between coughers and the established healthy adult population (p = 0.40).

### CCEI

The scores for cough patients on the CCEI were consistently elevated compared with published values for a normal population but lower than values for a psychiatric out-patient population. The mean scores for phobic anxiety, obsession, somatisation and depression subscales for cough patients were significantly higher than the means for published healthy controls (table 3) [9]. Correlation coefficients between the individual subscales on the CCEI suggested good internal consistency with those sharing common diagnostic criteria i.e. phobic anxiety and free floating anxiety correlating well (r = 0.635, p < 0.01).

### Correlation between psychological questionnaires

Pearson correlation coefficients between HAD anxiety subscale and STAI state anxiety and trait anxiety were highly significant (0.621 and 0.607 respectively, p < 0.01) suggesting strong correlation between questionnaires and good concurrent validity.

Correlation between the CCEI and other psychological questionnaires were highly significant for common diagnostic criteria indicating strong concurrent validity (free floating anxiety and HAD anxiety, r = 0.867, phobic anxiety and HAD anxiety, r = 0.603, phobic anxiety and trait anxiety, r = 0.582, CCEI depression and HAD depression, r = 0.633, p < 0.01 for all correlations).

Individuals with idiopathic cough had significantly higher state anxiety scores compared with those where a cause was identified and successfully treated. There was no significant difference between these two groups on any of the other psychoneurotic scales. No significant differences were seen between male and female cough patients. Similarly, patients reporting stressful situations as a precipitant for their cough did not score significantly differently on the questionnaires. There was weak positive correlation between cough symptom duration and both HAD depression and trait anxiety (0.321 and 0.320 respectively, p < 0.05).

## Discussion

Patients with persistent cough referred to a specialist cough clinic appear to have higher levels of emotional distress than would be expected in a healthy population. Apart from higher levels of state anxiety, there are no major distinguishing features in psychomorbidity between idiopathic coughers and individuals with successfully treated cough. Cough duration has some positive correlation with both anxiety and depression although age and gender appear to bear no relationship to the occurrence of psychiatric morbidity.

The level of anxiety disorder identified in this study is greater than the expected lifetime prevalence for anxiety disorders in the community, which has been estimated at 15% [12]. In particular for trait anxiety, 48% of cough patients in our study scored in the moderate and high range. The strong correlation between the anxiety subscales for both HAD and STAI questionnaires add particular validity to this finding. While Ludviksdottir and colleagues suggested a significant association between habitual coughing and anxiety, their patient group was selected on the basis of a positive response to questions concerning coughing, from a larger cohort of participants in the European Community Respiratory Health Survey [2]. Such a population is likely to differ considerably from individuals with persistent cough referred for specialist evaluation.

Using the CCEI questionnaire, the scores for almost all psychoneurotic symptoms measured in patients with chronic cough were significantly higher than scores in the healthy population but lower than scores in the psychiatric outpatients reported by Crown and Crisp [9]. In particular, the CCEI suggested high levels of phobic anxiety among coughers which concurred with the HAD and STAI questionnaires. The CCEI also identified increased levels of somatisation among our cough patients, which is consistent with reports of significantly higher somatization scores among cough patients compared to asymptomatic adults [13]. In a large, three centre study, which reported on lifetime prevalence of specific psychiatric disorders, somatization was very rare with a prevalence rate of less than 0.2% [12].

There are a number of explanations for our current findings. Firstly, it is known that persistent cough impacts negatively on the individuals' quality of life [1]. Patients with chronic cough suffer significant lifestyle and social restrictions and this may induce a psychological stress response. Secondly, the specific psychological profile of patients may influence their perception of symptoms. Patients with anxiety, depression and hypochondriasis are more aware of their body's physiology, for example their own heartbeat [14]. Increased levels of anxiety and somatization have also been associated with increased reporting of minor pain such as headache and abdominal pain [15]. Therefore it is possible that the general psychomorbidity associated with persistent cough might influence an individuals' awareness of the symptom and lowers the threshold for seeking medical attention.

The levels of emotional distress in particular anxiety among our cough patients are similar to that reported in patients with other chronic respiratory diseases [16]. However, the range and severity appear to be less than that identified in severe airways disease such as difficult-to-control asthma. We have recently reported that almost half of the patients with difficult asthma referred for specialist evaluation had a psychiatric diagnosis (depression in 60% of cases) identified at formal psychiatric assessment [17]. This high prevalence of depression has also been reported in patients referred for evaluation of a chronic cough [4].

Although our cough patients were carefully characterised, there are a number of limiting factors to our study. Significant differences in psychomorbidity between idiopathic coughers and successfully treated cough patients may have been overlooked because of the relatively small numbers in each group. Secondly, while comparison of the measures of psychomorbidity used in this study and measures of cough specific health status would have been of interest, participants were recruited prior to the publication of existing cough specific quality of life questionnaires [18,19]. Finally, given the cross-sectional design of this study, psychological questionnaires were only completed at initial presentation, and although changes in questionnaire scores over time would have been of interest, this was not an objective of the current study.

In summary, the findings from this study suggest that patients referred for evaluation of chronic cough have significant psychological distress. Failure to identify this may contribute to the slow response to specific therapy reported by clinicians [5]. While the use of self-assessment psychological questionnaires is not likely to discriminate individuals with idiopathic cough, it may identify those with high levels of emotional distress who could benefit from psychotherapy.

**Table 2:** Comparison of psychoneurotic scales between idiopathic cough patients (n = 15) and successfully treated patients (n = 42)

	***Treated cough (n = 42)***	***Idiopathic (n = 15)***	***Unpaired t value***	***P value ***
***HAD***				
***Anxiety***	6.74 (4.66)	5.27 (3.62)	- 1.107	0.136
***Depression***	3.81 (3.72)	3.93 (3.97)	0.109	0.457
***STAI***				
***State***	30.92 (8.20)	36.5 (9.53)	1.975	0.027*
***Trait***	38.92 (12.16)	39.15 (8.58)	0.063	0.475
***CCEI***				
***FFA***	5.47 (4.37)	6.75 (4.20)	0.859	0.197
***PA***	4.05 (3.34)	4.33 (3.60)	0.249	0.402
***OBS***	6.58 (3.76)	7.58 (3.11)	0.837	0.203
***SOM***	5.92 (3.98)	4.75 (4.48)	-0.086	0.196
***DEP***	4.71 (3.52)	4.50 (3.23)	1.49	0.07

Values given as mean (SD)* p < 0.05, **HAD **– Hospital Anxiety and Depression scale, **STAI **– State-Trait Anxiety Inventory, **CCEI **– Crown Crisp experiential Index,**FFA **– free floating anxiety, **PA **– phobic anxiety, **OBS **– obsession, **SOM **– somatisation, **DEP **– depression

**Table 3:** Comparison of mean scores on CCEI subscales for cough patients (n = 15) and published healthy controls(n = 109) [9]

	**All Cough (n = 57)**	**Published controls (n = 109) **[9]	**t value**	**p value**
***FFA***	5.8 (4.5)	5.11 (3.1)	0.96	N.S
***PA***	4.1 (3.4)	2.9 (2.2)	2.32	< 0.05
***OBS***	6.8 (3.6)	5.8 (3.1)	1.71	< 0.05
***SOM***	5.6 (4.1)	3.2 (2.4)	3.9	< 0.001
***DEP***	4.7 (3.4)	3.3 (2.3)	2.54	< 0.05

Values given as mean (SD)

**CCEI **– Crown Crisp experiential Index,**FFA **– free floating anxiety, **PA **– phobic anxiety, **OBS **– obsession,

**SOM **– somatisation, **DEP **– depression, **NS **– not significant
